# Experiences of UK clinical scientists (Physical Sciences modality) with their regulator, the Health and Care Professions Council: results of a 2022 survey

**DOI:** 10.1186/s12913-024-10956-7

**Published:** 2024-05-16

**Authors:** Mark McJury

**Affiliations:** University of Glasgow, Level 2, ICE Building, Queen Elizabeth University Hospital Campus, 1345 Govan Road, Glasgow, G51 4TF UK

**Keywords:** HCPC, Regulation of professions, Clinical scientists, Medical physicists, Survey

## Abstract

**Background:**

In healthcare, regulation of professions is an important tool to protect the public. With increasing regulation however, professions find themselves under increasing scrutiny. Recently there has also been considerable concern with regulator performance, with high profile reports pointing to cases of inefficiency and bias. Whilst reports have often focused on large staff groups, such as doctors, in the literature there is a dearth of data on the experiences of smaller professional groups such Clinical Scientists with their regulator, the Health and Care Professions Council.

This article reports the findings of a survey from Clinical Scientists (Physical Sciences modality) about their experiences with their regulator, and their perception of the quality and safety of that regulation.

**Methods:**

Between July–October 2022, a survey was conducted via the Medical Physics and Engineering mail-base, open to all medical physicists & engineers. Questions covered typical topics of registration, communication, audit and fitness to practice. The questionnaire consisted of open and closed questions. Likert scoring, and thematic analysis were used to assess the quantitative and qualitative data.

**Results:**

Of 146 responses recorded, analysis was based on 143 respondents. Overall survey sentiment was significantly more negative than positive, in terms of regulator performance (negative responses 159; positive 106; significant at *p* < 0.001). Continuous Professional Development audit was rated median 4; other topics were rated as neutral (fitness to practice, policies & procedures); and some as poor (value).

**Conclusions:**

The Clinical Scientist (Physical Sciences) professional registrants rated the performance of their regulator more negatively than other reported assessments (by the Professional Standards Authority). Survey respondents suggested a variety of performance aspects, such as communication and fitness to practice, would benefit from improvement. Indications from this small dataset, suggest a larger survey of HCPC registrants would be useful.

**Supplementary Information:**

The online version contains supplementary material available at 10.1186/s12913-024-10956-7.

## Background

**Fig. 1:**
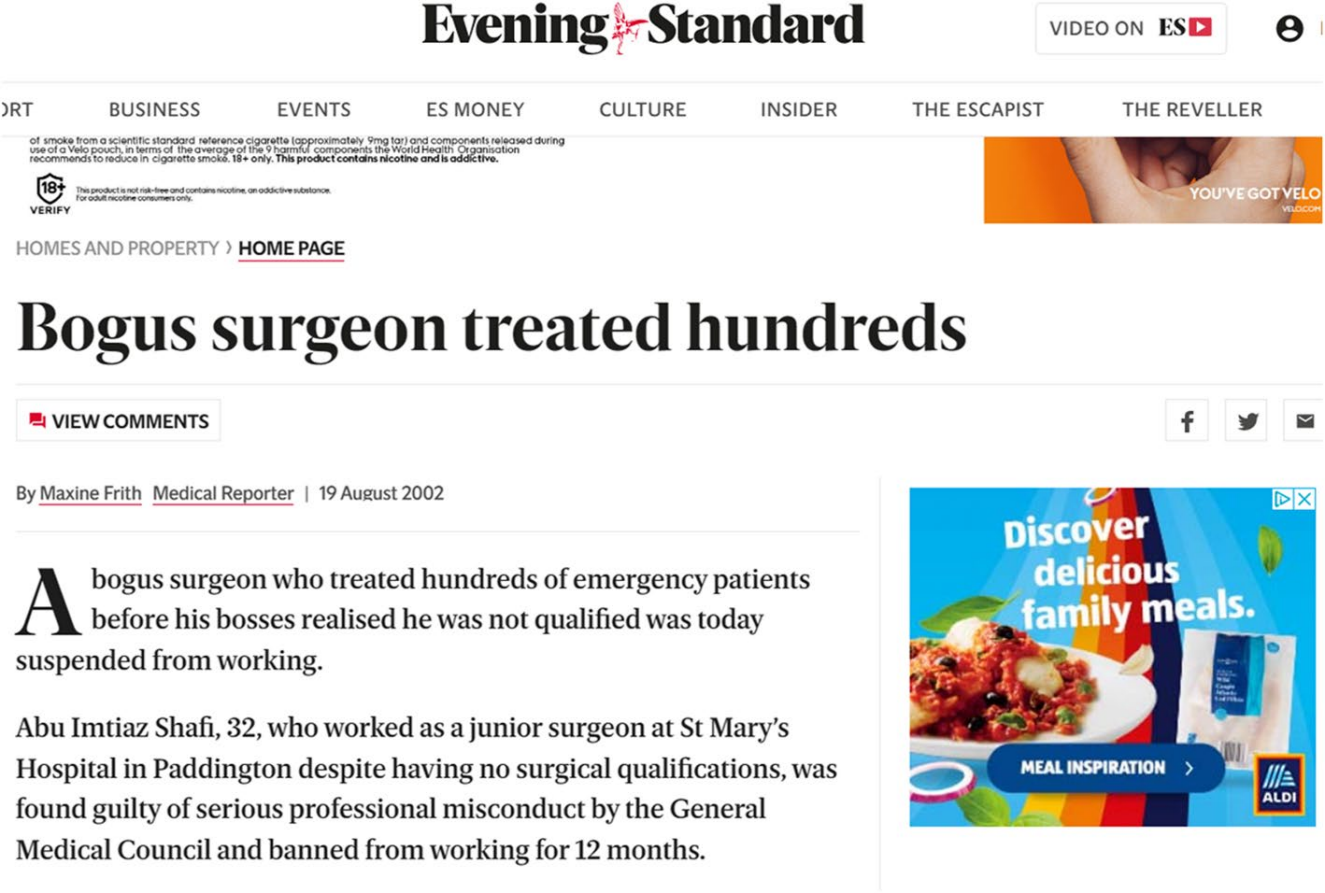
Recent UK media report on a bogus healthcare practitioner [2]

In Healthcare, protection of patients and the public is a core principle. Part the framework of protections, includes regulation of professions [1]. This aims to mitigate risks such as the risk from bogus practitioners – insufficiently trained people acting as fully-trained professional practitioners, see Fig. 1.

Regulation of professions ensures that titles (e.g. Doctor, Dentist, Clinical Scientist) are protected in law. The protected title means someone may *only* use that title, if they are on the national register, managed by the regulator – the Health and Care Professions Council (HCPC). It is a criminal offence to use a protected title if you are not entitled to do so [3]. There are a large number of regulators in healthcare – see Table 1. Most of the regulators manage a register for one profession, except the HCPC which regulates 15 professions.

**Table 1:** UK Healthcare regulators

Regulator	Role (UK) assoc. with fee level	2022 Annual registration fee /£
General Chiropractic Council	Chiropractor	800
General Dental Council	Dentist	690
General Osteopathic Council	Osteopath	570
General Medical Council	Licensed doctor	420
Pharmaceutical Society of Northern Ireland	Pharmacist	398
General Optical Council	Dispensing optician	360
General Pharmaceutical Council	Pharmacist	257
Nursing and Midwifery Council	Nurse/Midwife	120
**Health and Care Professions Council**	**Registrant (15 professions)**	**98.12**
Social Work England	Social Worker	90
Scottish Social Care Council	Social Worker	80
Social Care Wales	Social Worker	80
NI Social Care Council	Social Worker	65

Fees generally reported for one role & exclude any fee reductions

To be included on the register, a candidate must meet the regulators criteria for knowledge and training, and a key element to remain, is to show evidence of continuous professional development (CPD). Being on the register ensures that a practitioner has met the appropriate level of competence and professional practice.

For many healthcare workers, being on the HCPC register is a compulsory requirement to be appointable to a post. They must pay the necessary annual fees, and abide by the policies drawn-up by the regulator, and generally professions have no choice of regulator – these are statutory bodies, setup by government.

Recently, there has been considerable public dissatisfaction with the activity & performance of some regulators, notably *Ofwat* [4], and *Ofgem* [5]. Healthcare workers should expect a high level of professionalism, efficiency, and integrity from a regulator, as the regulator’s performance directly affects staff and public safety.

In terms of the regulation of UK Clinical Scientists, there is a dearth of data regarding experiences with the HCPC and views on the quality of regulation provided.

Findings are reported here from a 2022 survey of Medical Physicists and Engineers (one of the 16 job roles or ‘modalities’ under the umbrella of Clinical Scientist). The research aim was to assess experiences, and the level of ‘satisfaction’ with the regulator. For the remainder of this report, the term Clinical Scientist will be taken to mean Clinical Scientist (Medical Physicist/Engineer). The survey was designed to gather & explore data about opinions and experiences regarding several key aspects of how the HCPC performs its role, and perception of the quality & safety of regulation delivered.

## Methods

A short survey questionnaire was developed, with questions aimed to cover the main regulatory processes, including registration & renewal, CPD audit, and fitness-to-practice. There were also questions relating more generally to HCPC’s performance as an organisation, e.g. handling of personal data. Finally, participants were asked to rate the HCPC’s overall performance and what they felt was the ‘value’ of regulation. The survey questions are listed in the Supplementary file along with this article.

Questions were carefully worded and there was a balance of open and closed questions. A five-point Likert score was used to rate closed questions. The survey was anonymous, and the questions were not compulsory, allowing the responders to skip irrelevant or difficult questions. The survey also aimed to be as short & concise as possible, to be a minimal burden to busy clinical staff & hopefully maximise response rate. There were a small number of questions at the start of the survey, to collect basic demographics on the respondents (role, grade, UK nation etc.).

The survey was advertised on the online JISC-hosted UK Medical Physics and Engineering (UKMPE) mail-base. This offered convenient access for the majority of Clinical Scientists. The survey was advertised twice, to allow for potential work absence, holiday/illness etc. It was active from the end of July 2002 until October 2022, when responses appeared to saturate.

The data is a combination of quantitative rating scores, and qualitative text responses. This allows a mixed-methods approach to data analysis, combining quantitative assessment of the Likert scoring, and (recursive) thematic analysis of the free-text answers [6]. Thematic analysis is a standard tool, and has been reported as a useful & appropriate for assessing experiences, thoughts, or behaviours in a dataset [7]. The survey questions addressed the main themes, but further themes were identified using an inductive, data-driven approach. Qualitative data analysis (QDA) was performed using NVivo (QSR International).

Two survey questions attempted to obtain an overall perception of HCPC’s performance: the direct one (Q12), and a further question’Would you recommend HCPC as a regulator…?’. This latter question doesn’t perhaps add anything more, and in fact a few respondents suggested it was a slightly awkward question, given professions do not have a choice of regulator – so that has been excluded from the analysis.

Study conduct was performed in accordance with relevant guidelines and regulations [8, 9]. Before conducting the survey of Clinical Scientists, the survey was sent to their professional body, the Institute of Physics and Engineering in Medicine (IPEM). The IPEM Professional Standards Committee reviewed the survey questions [10]. Written informed consent was obtained from participants.

## Data analysis

Data was collected via an MS form, in a single excel sheet and stored on a secure network drive. The respondents were anonymised, and the data checked for errors. The data was then imported into NVivo v12.

Qualitative data was manually coded for themes, and auto-coded for sentiment. An inductive approach was used to develop themes.

The sample size of responses allowed the use of simple parametric tests to establish the level of statistical significance.

## Findings

### Survey demographics

A total of 146 responses were collected. Two respondents noted that they worked as an HCPC Partner (a paid role). They were excluded from the analysis due to potential conflict of interest. One respondent’s responses were all blank aside from the demographic data, so they were also excluded from further analysis.

**Fig. 2:**
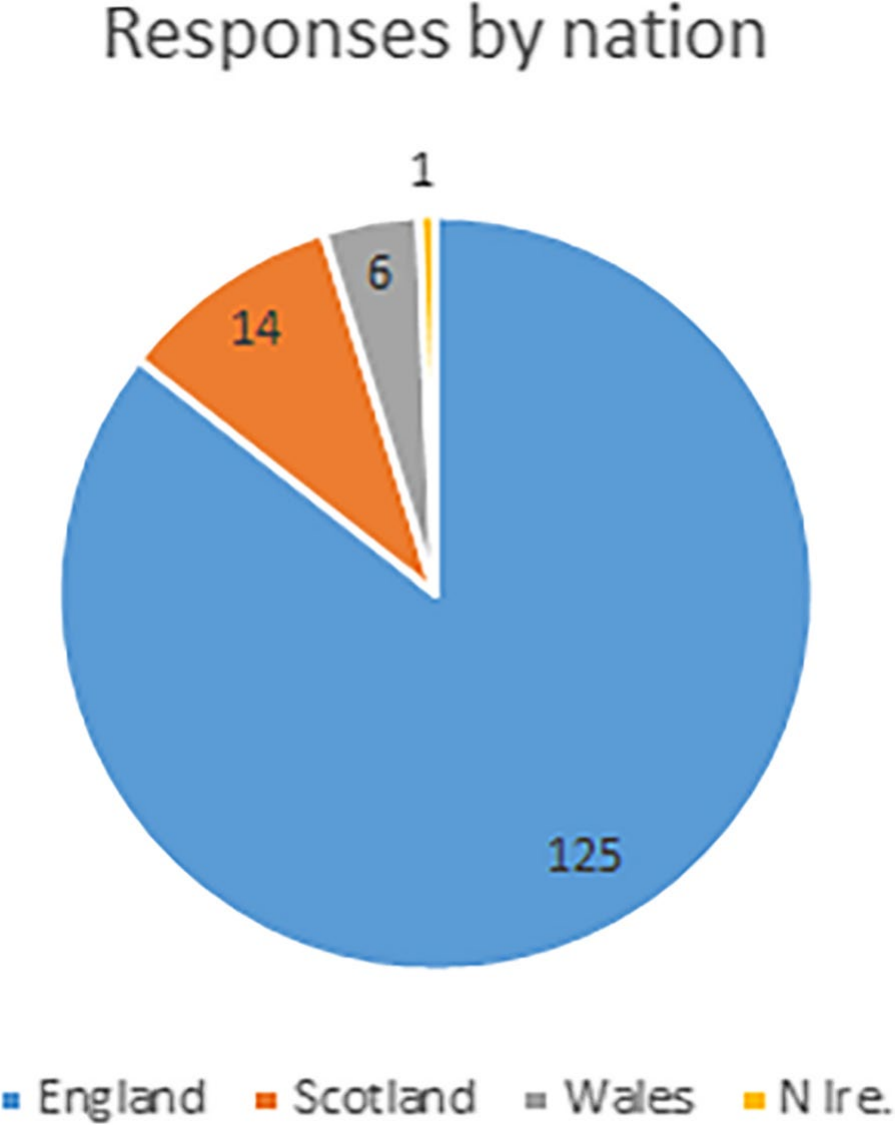
Proportion of respondents, by nation

Analysis is based on 143 responses, which represents ~ 6% of the UK profession [11]. It is arguable whether it is representative of the profession at this proportion of response – but these responses do offer the only sizeable pool of data currently available. The survey was aimed at those who are on the statutory register as they are most likely to have relevant interactions & experiences of the HCPC, but a small number of responses were also received from Clinical Technologists (Medical Technical Officers-MTOs) and Engineers (CEs) and these have been included in the analysis. Figure 2 shows the breakdown in respondents, by nation.

**Fig. 3:**
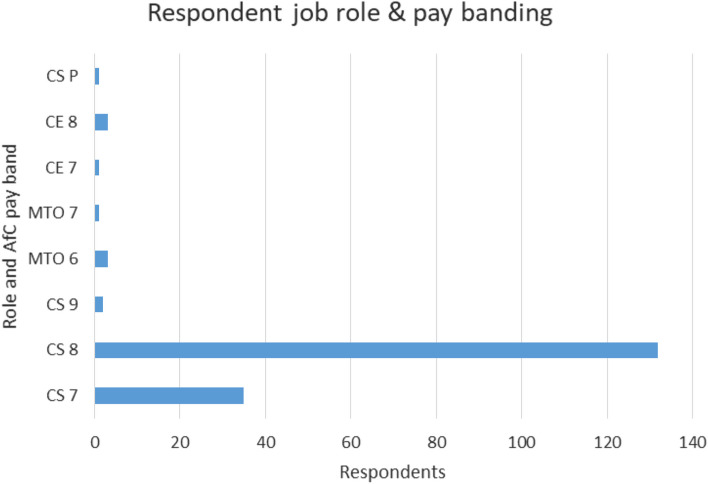
Breakdown in respondents, by role and pay banding

Of the respondents, 91% are registered Clinical Scientists, and would therefore have a broad range of experience with HCPC and its processes. Mean time on the register was 12 yrs. Respondents show a large range in seniority, and their roles are shown in Fig. 3 (CS-Clinical Scientist; CE-Clinical Engineer; MTO-Medical Technical Officer/Technician; CS-P are those working in private healthcare settings, so not on Agenda for Change (AfC) pay bands).

These data can be compared with the most recent HCPC ‘snapshot’ of the CS registrants (find here: Registrants by profession snapshot—1967 to 2019 | (https://www.hcpc-uk.org/resources/data/2019/registrant-snapshot/)).

The perception of overall regulator performance, can be assessed in two ways – one interview question directly asked for a rating score, and the overall survey sentiment also offers additional insight.

The score for overall performance was a median of 3 (mean 2.7; response rate 90%) which suggests neutral satisfaction.

Respondents were not asked directly to explain this overall performance rating – themes were extracted from the questionnaire as a whole.

The auto-coded sentiment scores generated in the NVivo software are shown in Table 2. There is a significantly stronger negative sentiment than positive for HCPC performance – moderate, strong and total sentiment scores are all higher for negative sentiment. The normal test for a single proportion (109), shows the negative and positive sentiment differences have statistical significance with *p* < 0.001. Whilst the PSA assessment of HCPC performance in 2022–23 shows 100% performance for 4 out of 5 assessment areas, survey data here from regulated professionals suggests considerably less satisfaction with HCPC. This raises associated questions about the relevance and validity of PSA assessment.

**Table 2:** NVivo-generated sentiment analysis scores

Survey Sentiment scores		
**Themes**	**Responses**	**Total**
Very positive	43	106
Moderately positive	63	
Moderately negative	103	159
Very negative	56	

A large number of respondents seem to question the value of regulation. Whilst many accepted the value for it in terms of protecting the safety of the public, many questioned its relevance & benefit to themselves. Many respondents also queried the payment model where although the main beneficiaries of regulation are the public & the employer, it is the registrants actually pay the fees for registration. There was very little mention in survey responses, of benefit in terms of protected-title. These issues were amalgamated into Theme 1—*Value of regulation*, with the two sub-themes *Value in monetary terms* (value-for-money) and *Value in professional terms* (benefit and relevance to the individual professional) (see Table 3).

**Table 3:** Final themes and sub-themes

	***Theme***	***Sub-theme***
***1***	***Value of regulation***	*Value in monetary terms (inc. fees & HCPC spending)*
		*Value in professional terms (inc. relevance *etc*.)*
***2***	***HCPC organisational performance***	*Communication & handling data*
		*Registration and Renewal*
		*Engagement with the profession*
		*Audit process & policies*
***3***	***Registrant competence and vulnerability***	
***4***	***Suggestions for improvement***	

In the survey, several aspects of HCPC organisational performance were scored – handling of personal data, registration and renewal, engagement with the profession, audit, and the quality and usefulness of HCPC policies. These formed Theme 2 and its sub-themes.

A third theme *Registrant competence and vulnerability*, was developed to focus on responses to questions related to the assessment of registrant competence and Fitness To Practice (FTP) processes.

Finally, the survey also directly asked respondents if they could suggest improvements which would have resulted in higher scoring for regulation quality and performance. These were grouped into Theme 4.

### Theme 1 – Value of regulation

#### Value in monetary terms

The Likert score for value-for-money was a median of 2 (mean 2.3; response rate 100%) which suggests dissatisfaction. This is one of the few survey questions to elicit a 100% response rate – a clear signal of its importance for registrants.

There was a high number of responses suggesting fees are too expensive (and a significantly smaller number suggesting good value). This ties in with some respondents explaining that the ‘benefit’ from registration is mainly for the employer (an assurance of high quality, well-trained staff). Several respondents point to little ‘tangible’ benefit for registrants and query whether the payment model is fair and if the employer should pay registrant fees.

“Expensive fees for what appears to be very little support.” Resp094

“It seems that I pay about £100 per year to have my name written on a list. It is unclear to me what the HCPC actually does in order to justify such a high fee.” Resp014

*“I get, quite literally, nothing from it. It’s essentially a tax on work.” Resp008*

Several respondents suggested that as registration was mandated by the employer, it was in essence an additional ‘tax’ on their employment, which was highlighted previously by Unison [12]. A comparator for payment model, are the checks preformed on potential staff who will be working with children and vulnerable adults. In general, these ‘disclosure’ checks are paid for by the employer, however the checks are not recurrent cost for each individual, but done once at recruitment.

#### Value in professional terms & relevance

This was not a direct question on the questionnaire, but emerged consistently in survey responses. Aside from value-for-money, the value of regulation can also refer to more general benefit and relevance for a professional, for example in protecting a professional title or emphasising the importance of a role. Many respondents commented, in relation to the ‘value’ of regulation, about the *relevance* of the HCPC to them and their job/role.

The largest number of responses highlighted the lack of clarity about HCPC’s role, and also to note its lack of relevance felt by a significant proportion of respondents.

“Not sure I have seen any value in my registration except that it is a requirement for my role” Resp017

“I really fail to understand what (sic) the benefits of registration.” Resp018

“They do not promote the profession. I see no evidence of supporting the profession. I pay to have the title and I am not aware of any other benefits.” Resp038

### Theme 2 – HCPC performance

#### Communication & handling data

The survey questionnaire did not have a specific question relating to communication, therefore no specific Likert scores are available. Rather, communication was a sub-theme which emerged in survey responses. The response numbers related to positive (1) and negative experiences (50) clearly suggest an overall experience of poor communication processes (and statistically significant at *p* < 0.001 for a normal proportion test).

One respondent noted they had ‘given up’ trying to communicate with HCPC electronically. Several respondents also noted issues with conventional communication—letters from HCPC going to old addresses, or being very slow to arrive.

“…I have given up on contacting by electronic means.” Resp134

When trying to renew their registration, communication with HCPC was so difficult that two respondents noted they raised a formal complaint.

A number of respondents noted that when they eventually got through to the HCPC, staff were helpful, so the main communication issue may relate to insufficiently resourced lines of communication (phones & email) or the need for a more focussed first point of contact e.g. some form of helpdesk or triaging system.

“Recently long wait to get through to speak to someone… Once through staff very helpful.” Resp126

This topic overlaps with the next (Processing Registration & renewals) in that both involve online logins, website use etc.

Security & data handling was rated as neutral (median 3, mean 3.4; response rate 91%). Although responses were balanced in terms of satisfaction, a significant number noted a lack of knowledge about HCPC processes. There are almost equal proportions of respondents reporting no issues, some problems with handling of personal data, or insufficient knowledge to express an opinion.

#### Registration and renewal

The score for processing registrations & renewals, was a median of 4 (mean 3.5; response rate 92%) which suggests modest satisfaction.

The overall rating also suggests that the issues may have been experienced by a comparative minority of registrants and that for most, renewal was straightforward.

*“They expected people to call their phone number, which then wasn’t picked up. They didn’t reply to emails except after repeated attempts and finally having to resort to raising a complaint.” Resp023*

“Difficult to get a timely response. Difficult to discuss my situation with a human being…” Resp044

Although the Likert score is positive, the themes in responses explaining the rating, are more mixed. Many respondents mentioned either having or knowing others who had issues with registration renewal, and its online processes including payments. A few respondents mentioned that the process was unforgiving of small errors. One respondent, for example, missed ticking a box on the renewal form, was removed from the register and experienced significant difficulties (poor communication with HCPC) getting the issue resolved.

Some respondents noted issues related to a long absence from work (e.g. maternity/illness etc.) causing them to miss registration deadlines – for some, this seems to have resulted in additional fees to renew registration. It seems rather easy for small errors (on either side) to result in registrants being removed from the register. For registrants, this can have very serious consequences and it can then be difficult and slow to resolve this, sometimes whilst on no pay. There have also been other reported instances of renewal payment collection errors [13].

*“I had been off work… and had missed their renewal emails…I was told that there would be no allowances for this situation, and I would have to pay an additional fee to re-register…” Resp139.*

Some respondents raised the issue of exclusion – certain staff groups not being included on the register—such as Clinical Technologists and Clinical Engineers. This desire for inclusion, also points to a perception of value in being on the register. One respondent raised an issue of very difficult and slow processing of registration for a candidate from outside the UK.

“Staff member who qualified as medical physicist abroad…has had a dreadful, drawn out and fruitless experience.” Resp135

Overall, many respondents noted difficulties in renewing registration and issues with HCPC’s online processes. Some of these issues (e.g. website renewal problems) may have been temporary and are now resolved, but others (e.g. available routes for registration) remain to be resolved.

#### Audit process & policies

In the survey, 12% respondents reported having been audited by HCPC regarding their CPD (response rate 97%). This is well above the level of 2.5% of each profession, which HCPC aims to review at each renewal [14], and similar values reported by some professional bodies [15]. The participants seem representative, although two respondents mentioned their perception of low audit rates. Data on CPD audit is available here: https://www.hcpc-uk.org/about-us/insights-and-data/cpd/cpd-audit-reports/

Respondents rated the process of being audited as a median of 4 (mean 3.7), which is the joint highest score on the survey, pointing to satisfaction with the process. From the responses, the overall perception could be summed up as straight-forward, but time-consuming. Without regular record-keeping, unfortunately most audits will be time-consuming – the HCPC more so, as it is not an annual audit, but covers the two preceding years.

Some respondents did find the process not only straight-forward, but also useful (related to feedback received). However, responses regarding feedback were mixed, with comments on both good, and poor feedback from HCPC.

“Not difficult but quite long-winded” Resp008

“Very stressful and time consuming” Resp081

“While it was a lot of work the process seemed very thorough and well explained.” Resp114

The HCPC’s policies & procedures were rated as a median of 3 (mean 3.2; response rate 98%). This neutral score could suggest a mixture of confidence in HCPC practise. This score may also reflect the fact that the majority of respondents had either not read, or felt they had no need to read the policies, and so are largely unfamiliar with them.

The reasons for this lack of familiarity are also explained by some respondents – four commented that the policies & procedures are rather too generic/vague. Three respondents noted that they felt the policies were not sufficiently relevant to their clinical roles to be useful. This may be due to the policies being written at a level to be applicable to registrants from all 16 modalities – and perhaps a limitation of the nature of HCPC as a very large regulator. Familiarity seemed mainly to be restricted to policies around registration, and CPD. There were slightly lower response levels for positive sentiment (6), than negative sentiment (9).

“I’ve never had cause to read them.” Resp115

“Detached from the real clinical interface for our professions…” Resp083

HCPC split their policies into ‘corporate’- which relate to organisational issues (e.g. equality & diversity; find them here: Our policies and procedures | (https://www.hcpc-uk.org/about-us/corporate-governance/freedom-of-information/policies/#:~:text=Our%20main%20policies%20and%20procedures%201%20Customer%20feedback,scheme%20...%207%20Freedom%20of%20Information%20Policy%20)) and those more relevant to professions (e.g. relating to the register; find them here: Resources | (https://www.hcpc-uk.org/resources/?Query=&Categories=76)).

One respondent noted not only that the policies were ‘as you might expect’, but felt the policies were less demanding than those from other similar bodies such as the CQC (https://www.cqc.org.uk/publications).

“…Other regulatory bodies (such as the CQC for example) have policies and procedures that are a lot more challenging to comply with.” Resp022

### Theme 3 – Registrant competence and vulnerability

In this survey, 3.5% (5/143) of respondents noted some involvement with the HCPC’s Fitness to Practice service. These interactions were rated at a median of 3 (mean 2.8) suggesting neutral sentiment.

Firstly, we can immediately see the level of interaction with the FTP team is very small. CS registrants represent approx. 2% of HCPC registrants, and the level of CS referrals to FTP in 2020–21 was 0.2% [16].

The data is a very small sample, but responses vary strongly, so it is worth digging a little further into the granularity of individual responses. Response scores were 1, 1, 2, 5, 5 – which are mainly at the extremes of the rating spectrum. The majority of respondents described poor experiences with the FTP team: errors, a process which was ‘extremely prolonged’, involved slow/poor communication, and processes which were ‘entirely opaque’.

“It is slow, the process was badly managed… and the system was entirely opaque,” Resp37

*“They were hard to contact and I didn't feel they listened…no explanation, apology or assurance it would not happen again. It left my colleague disillusioned and me very angry on their behalf…” Resp044*

**Fig. 4:**
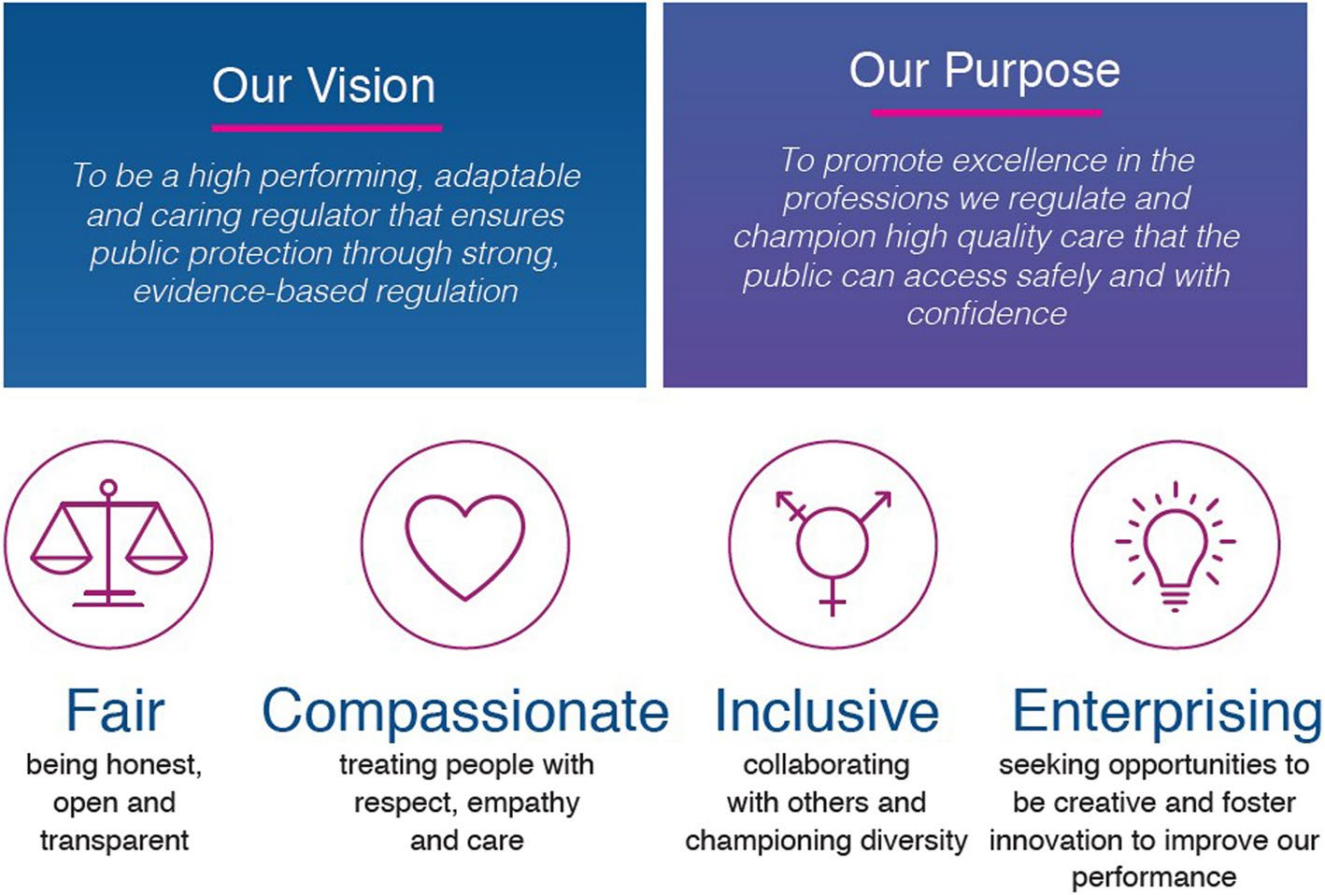
HCPC’s vision statement & purpose [17]

Some respondents commented that the team were not only difficult to contact, but also didn’t seem to listen. At the end of a process which involved errors from HCPC, one respondent noted were ‘no explanation, apologies or assurance that it would not happen again’, leaving the registrant ‘disillusioned’. These experiences do not fit with the HCPC’s stated goal to be a compassionate regulator, see Fig. 4. Arguably it is more difficult to change a culture of behaviour and beliefs, than to publish a corporate goal or statement of vision.

Some survey respondents have noted the necessity of regulation for our profession.

“Ultimately I am very grateful that I can register as a professional.” Resp024

### Theme 4 – Suggestions for improved regulation

Following the question relating to overall performance, respondents were invited to suggest things which might improve their rating for HCPC’s performance and value. These suggestions were also combined with those which appeared in earlier survey responses.

#### Benefits

Although we are in a current cost-of-living crisis, responses did not query simply high *absolute* cost of fees, but also queried the value/benefit of HCPC regulation for registrants. Many responses expressed doubt as to the added value & relevance of HCPC registration for them. They seem to point to a desire for more *tangible* benefit from their fees. Perhaps, given the costs and levels of scrutiny, registrants want some definite benefit to *balance the scales*.

“Cost less and do more for the people who are on the register.” Resp089

“Vastly reduced cost. Employer paying registrant fees.” Resp074

A significant number of responses pointed out that the main benefits of registration are for the public, and for employers – but that it is the registrants who pay for registration. Many queries why this should be, and whether there should be a different payment model, where for example employers pay.

Similarly, some respondents felt that the HCPC’s unusual position of regulating a large swathe of healthcare professions was not necessarily helpful for their profession or others.

Communication and response times are obviously an issue of concern for registrants, and improvements are needed based on the low satisfaction levels reported here. This is also linked to a wish for increased engagement with the CS profession.

“Engagement with the workforce, specialism specific development, reduced fees” Resp025

Some responses suggested they would be comforted by increased accountability / governance of HCPC including improved FTP efficiency.

“More accountability to registrants” Resp130

Finally, improvement in terms of additional registration routes for Engineers & Technical staff were also suggested. It may be damaging to work-place moral, if two professionals doing roles of a similar nature are not being governanced is the same way and if there is not parity of their gross salary due to mandatory professional fees & reductions.

## Discussion

### Theme 1 – Value of regulation

*Value-for-money*: This will vary between individuals depending on many variables, such as upbringing & environment, salary, lifestyle priorities, political persuasion, and so on. However, many of these factors should balance in a large sample. In general, it can be suggestive of satisfaction (or lack of) with a service. The score here suggesting dissatisfaction, echoes with other reports on HCPC’s spending, and financial irregularities [18, 19].

In the survey findings, respondents have voiced dissatisfaction with registration value for money. In fact, HCPC’s registration fees are not high when compared to the other healthcare professions regulators. Table 1 shows data from 2021–22 for regulator annual registration fees. However, the HCPC has risen from having the lowest regulator fees in 2014–5, to its current position (9^th^ of 13) slightly higher in the table. Perhaps more concerning than the absolute level of fees, are when large increases are proposed [12, 20–22].

However, fees have regularly increased to current figure of £196.48 for a two-year cycle. During a consultation process in 2018, the Academy for Healthcare Clinical Scientists (AHCS) wrote an open letter to the HCPC, disputing what they felt was a disproportionate fee increase [23]. Further fee rises have also been well above the level of inflation at the time.

HCPC expenditure (which is linked to registration fees) has arguably been even more controversial than fee increases – noted by several respondents. A freedom of information (FOI) request in 2016 showed HCPC’s spending of £17,000 for their Christmas party [18] – which amounts to just over £76 per person. This cost was close to the annual registration fee (at that time) for registrants.

In 2019, regulation of social workers in England moved from HCPC, to Social Work England. This resulted in a loss of over 100,000 registrants, and a loss in registration fee income. HCPC raised fees to compensate, but a freedom of information (FoI) request in 2020 [18] showed that even though there was an associated lowering in workload associated with the loss of 100 k registrants, the HCPC had no redundancies, suggesting the loss of income was compensated mainly by the fees increase.

#### Inherent value & relevance

One of HCPC’s aims is to promote ‘the value of regulation’ [24]. However, not only is there dissatisfaction with value-for-money, the second highest response suggests a lack of inherent value (or benefit) from regulation to the individual registrant. In some ways, there is a lack of balance – registrants are under increasing scrutiny, but feel there is little direct benefit, to provide balance.

This also suggests that HCPC’s aim or message is not getting through to the CS profession. It’s not clear what the HCPC 2021–22 achieved milestone – ‘Embedded our registrant experiences research into employee learning and development and inductions’ has actually achieved.

A large number of responses pointed to the lack of clarity about HCPC’s role, and also to note its lack of relevance for respondents. Some of this is understandable – until recently, many CS registrants will have little interaction with HCPC. They would typically get one email reminder each year to renew their registration and pay those fees, and hear little else from the HCPC. That is beginning to change, and HCPC have recently begun to send more regular, direct emails/updates to registrants.

However, for many registrants, the HCPC appears not to be clearly communicating its role, or the relevance/importance of regulation. As mentioned above, this also links in to previous mentions of the lack of any tangible benefit for registrants. Some note little more relevance other than the mandatory aspects of regulation.

Finally, relevance is also queried in relation to the limited access for some professional groups to a professional register. The current situation of gaps in registration for some groups, results in two situations – firstly, for Clinical Scientists and Clinical Engineers/Technologists, one group has to compulsorily pay a fee to be allowed/approved to do their job and the other does not; also, the public are routinely helped and assisted by Clinical Scientists and Clinical Engineers/Technologists – but only one group is regulated to ensure public safety.

### Theme 2 – HCPC performance

#### HCPC Communication

This was highlighted by respondents as often poor. Recently in the media, there has been a concern raised by The College of Paramedics (CoP) about communications issues with HCPC—changes to the HCPC policy on the use of social media [25]. They raised particular concerns about the use of social media content and ‘historical content’ in the context of investigations of fitness-to practice.

There have previously been some concerns raised on the UKMPE mail-base regarding handling of personal data, and lack of efficiency in addressing the issue [26]. Several messages detailed HCPC communicating unencrypted registrant passwords in emails and sending personal data to the incorrect registrant. Some on the forum noted that they had reported this problem over a period of several years to HCPC, suggesting HCPC’s response to these serious issues was extremely slow. Several responses noted these previous issues.

#### Registration processes

Although responses here show some satisfaction, there have been reports in the media of significant issues with registration (such as removing registrants from the register in error) with associated impact for patients and the public [27, 28]. Similarly, there were reports on the UKMPE mail-base of significant issues with registration renewals being problematic [26]. In Scotland, NHS.net email accounts ceased to be supported in July-Sept 2020 and the associated lack of access to email accounts and messages used for HCPC communication and registration, caused a major issue in registration renewal. This coincided with COVID lockdowns and a period of unusually difficult communication with HCPC. If NHS staff lose registration (irrespective of the reason), respondents noted that some Human Resources (HR) departments were quick to suspend staff from work, and in some cases withhold pay. That spike in difficulties is likely the cause of the most common responses suggesting issues with a complicated process.

### Theme 3 – Registrant competence and vulnerability

In safe-guarding public safety, a key task for a healthcare regulator is assessing the competence of registrants. This is done via a small set of related activities. Registrants must return regular evidence of CPD, and these are audited for 2.5% registrants. This process is simple and routine, and as seen in *Theme 2* responses here suggest registrants are reasonably satisfied with this process.

More formal and in-depth competence assessment happens when a complaint is raised against a registrant, either by a work colleague/management, a member of the public or occasionally by the HCPC itself. The process is complex, lengthy and can end in a registrant attending a court hearing [29].

It is usual for registrants to continue in their normal job during FTP investigations – effectively the public remains at risk from a registrant if their competence is eventually proven to be below the regulators standards, so there is a need for investigations to be efficient both in timeliness, and outcome.

Obviously, being under investigation can be highly stressful, and has the potential for the registrant to be ‘struck off’ the register, and lose their job if registration is mandated (e.g. NHS posts). There are many reports of the process & experience either provoking or increasing underlying mental health challenges [30–32]. Along with efficiency, a regulator needs to behave compassionately. Investigations of highly-skilled professionals engaging in complex work activities, is also necessarily complex and requires a high degree of knowledge and experience from the regulator’s investigational panel.

The Professional Standards Authority (PSA) regulate the HCPC, and publish annual reviews of their performance (https://www.professionalstandards.org.uk/publications/performance-reviews) (see Table 4). HCPC performance as reported by PSA, seems to be generally higher than noted by survey respondents here. For 2022–23, aside from one area, the HCPC has scored 100% for performance, which seems at odds with these survey responses [33]. The FTP team is notable in repeatedly performing very poorly compared to most other sections of the HCPC (even though the majority of the HCPC budget goes to FTP activity, see Fig. 4). The HCPC Annual Report 2018–9 [34] highlighted the completion of the first phase of the Fitness-To-Practice Improvement Plan. This delivered “A root and branch review of this regulatory function… a restructure, tightened roles and processes and the introduction of a new Threshold Policy”, but this seems to have no impact on the performance reported by the PSA for the next few years shown in Table 4. However, the most recent data does suggest improvement, and HCPC continues to develop FTP team practice [17].

**Table 4:** HCPC’s performance, scored on the PSA’s Standards of good regulation

PSA Standards of good regulation—HCPC performance scores				
	2019–20	2020–21	2021–22	2022–23
General Standards	4/5	4/5	5/5	5/5
Guidance and Standards	2/2	2/2	2/2	2/2
Education and Training	2/2	2/2	2/2	2/2
Registration	4/4	4/4	3/4	4/4
**Fitness to Practise**	**1/5**	**1/5**	**1/5**	**3/5**

**Fig. 5:**
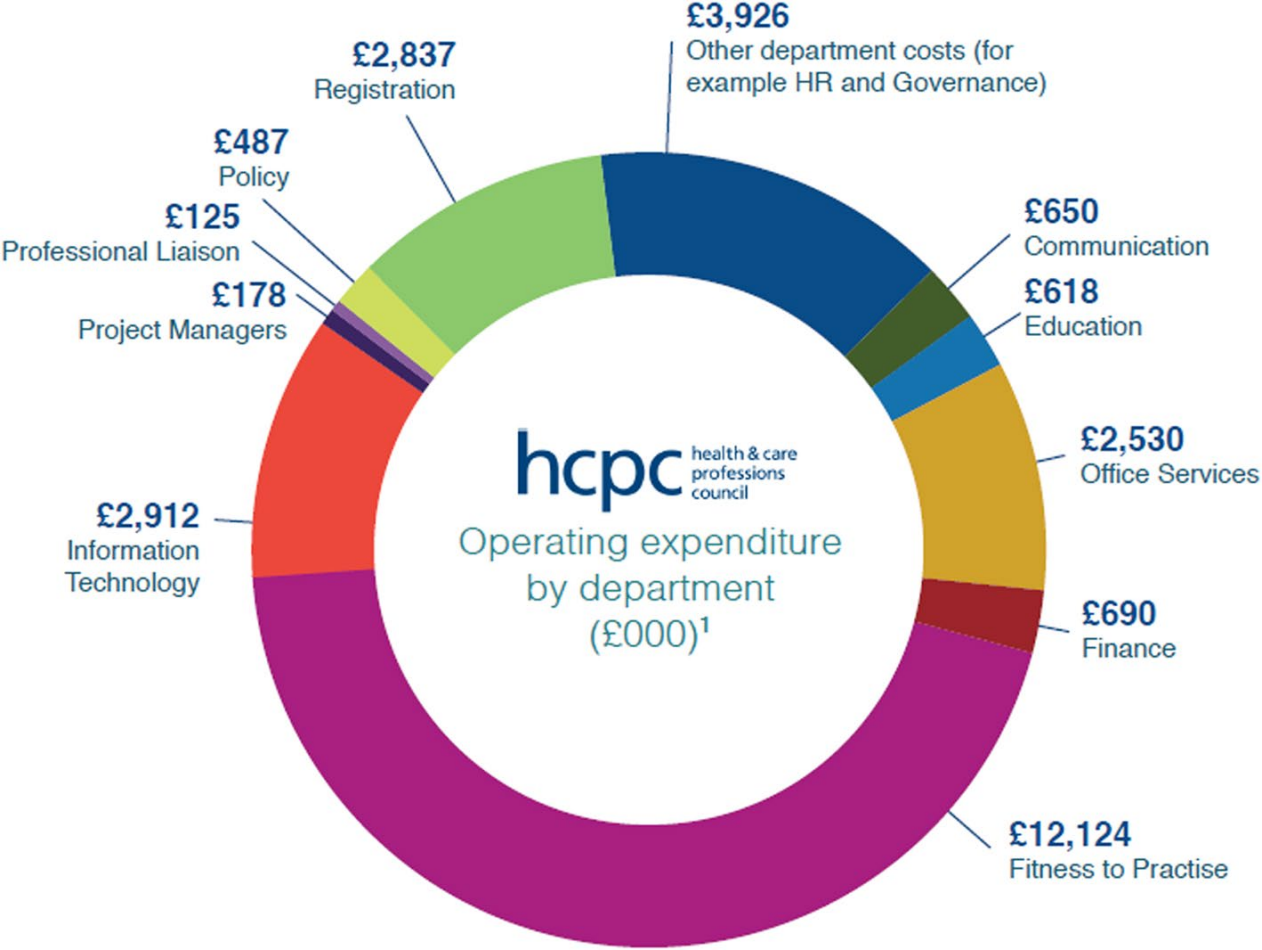
HCPC expenditure for the year 2020–21 [17]

There are other reports of poor experiences with this team [35, 36], and in one report the FTP team’s processes have been noted as being rather inhumane [35].

Regulation is an important part of public protection, but how effectively it is managed & enforced is also a concern, given it involves increased scrutiny of registrants. A topical comparator is the current dissatisfaction by a large section of the public about several other government regulators allowing seemingly poor performance to go unchecked [4, 5].

It is arguable, that registrants remain on the register as long as the HCPC allows them. Several respondents in this survey noted being removed from the register through HCPC administrative error. Removal could also happen through poor judgement/decision-making – the FTP team handle large numbers of very complex investigational cases – 1603 concluded cases for the year 2021–22 and 1024 hearings [16]. Every justice system is subject to a level of error – guilty parties can be erroneously ‘cleared’, and vice-versa. It is essential therefore, that policies & procedures relating to FTP are fit for purpose—that the FTP team work effectively and humanely, and that there is genuine & effective governance of HCPC to ensure accountability. In this survey, some respondents seem to be saying that currently this seems not to be the case.

### Theme 4 – Suggestions for improved regulation

It might have been anticipated that the greatest concern is costs, especially in the current cost-of-living crisis. The recent HCPC consultation to increase fees [37] seems particularly tone-deaf and has caused concern across the professions [21, 22].

#### Benefit

Above findings show respondents are interested in lower fees, but also increased benefit for their fees. Some respondents pointed out that whilst registrants pay for registration, benefit is mainly for the public and employers. The HCPC is a statutory body, its funding model will have been designed/decided upon by government, and may be unlikely to change. However, there are a variety of potential regulation models [38], and so change is possible. A review of the financial model for regulation may be welcome.

#### Regulator size

Some aspects of HCPC performance, policies, and distribution of spending, is related to the nature of it being the largest and only multi-professional regulator in the healthcare sector. Data from the HCPC suggests (see Fig. 5) that the majority of spending relates to FTP activity. Data also points to Clinical Scientists having very low levels of FTP investigation compared to others in HCPC [16]. This suggests that a significant proportion of CS registrant fees are used to investigate other professions. It’s possible (perhaps simplistically) that if, like many other healthcare professions such as doctors & dentists who’s regulator is concerned only with that single profession, if CSs were regulated separately, their registrant fees may be reduced. This model of single-profession regulation may also mitigate against other disadvantages of the HCPC’s practice, such as the ‘generic’ policies aiming to apply to a pool of 15 professions.

#### FTP

Although there is a very low level of data for this topic, the concerned raised by registrants are serious in nature. There also seems to be issues in handling of complaints related to this service and advocacy for registrants. Certainly, there is a clear governance path via PSA, to the Health Secretary. However, this does not offer a route for individual complaints to be raised and addressed. Unlike complaints from the public in other areas, there is no recourse to an ombudsman for registrants. The only option for individual registrants, is the submission of a formal complaint to the HCPC itself, which is dealt with internally. Comments from survey respondents suggest this process does not guarantee satisfaction. Indeed, one of the respondents who mentioned submitting a complaint, made it clear they remained unhappy with HCPC’s response. Overall, there seems to be a lack of clear & effective advocacy for registrants.

“…the HCPC’s stance appeared to be guilty until proven innocent… At no point did I feel the HCPC cared that their (sic) was an individual involved....” Resp044.

FTP processes affect a comparatively small number of CS registrants, compared to other professions. However, it seems clear that the majority of those who have interacted with the FTP team have had poor experiences, and respondents have suggested improvements are needed. The reason for FTP investigations, is protection of staff and the public. If processes are slow, and investigations prolonged, or decisions flawed, the public may be exposed to increased levels of risk, as healthcare practitioners who may be lacking in competence continue to practice. The data in Table 4 shows concerning but improving trends in FTP performance levels.

Finally, improvement in terms of additional registration routes for Engineers & Technical staff were also suggested. It may be damaging to work-place moral, if two professionals doing roles of a similar nature are not being governanced is the same way and if there is not parity of their gross salary due to mandatory professional fees & reductions.

### Limitations

There are two main limitations to this work. Firstly, due to time constraints, there was no pilot work done when designing the survey questionnaire. This may have helped, as noted earlier, a few responses pointed to some awkwardness with one survey question. Although no pilot work was done, the questionnaire was reviewed by the IPEM Professional Standards Committee, as noted in the Acknowledgements section.

The other obvious limitation is the low response rate (~ 6% of UK Medical Physicists). Circulation of the survey was performed via the only online forum for the profession currently available. The survey was advertised multiple times to ensure visibility to staff who may have missed it initially due to leave etc. However, the forum does reach 100% of the profession, and some addressees may have filters set to send specific posts to junk folders etc. The professional body IPEM declined to offer support in circulating the survey (believing the issues involved would affect/be of interest only to a small minority of members.)

The low response rate also has a particular impact on the pool of responses relating to FTP issues, which inherently affect low numbers of registrants.

However, the importance of some of the findings here (e.g. expressed dissatisfaction with regulation in terms of value; the poor experience of some members with the Registration, Communication and FTP teams) and the low sample surveyed, both justify the need for a larger follow-on survey, across all of Clinical Science.

## Conclusions

In Healthcare, regulation of professions is a key aspect of protecting the public. However, to be effective, regulation must be performed professionally, impartially, and associated concerns or complaints investigated efficiently and respectfully.

This report presents findings from a survey aimed at collecting a snap-shot of the experiences of Clinical Scientists with their regulator, and their perception of the quality and safety of that regulation performance.

Overall survey sentiment scores showed a significantly more negative responses than positive. Survey comments relate not only to current issues, but to previous problems and controversial issues [18, 26]. It seems that some respondents have at some point lost confidence and trust in the HCPC, and survey responses suggest there has not been enough engagement and work done by HCPC to repair and rebuild this trust.

In the midst of a cost of living crisis, costs are a large concern for many. The HCPC fees are neither the highest not lowest amongst the healthcare regulators. Spending is transparent, and details can be found in any of the HCPC’s annual reports.

A repeating sub-theme in responses, was a lack of tangible value for the registrant, and that the employer should pay the costs of registration, where registration is mandated by the job.

Many respondents have suggested that they feel there should be more proactive engagement from HCPC with the profession. Most respondents were not familiar with or felt the HCPC policies are relevant/important to them.

Survey data showed moderate satisfaction with registration processes for the majority of respondents. Some respondents also noted a lack of registration route for engineering & technical healthcare staff. CPD processes also achieved a score indicating registrant satisfaction. This generated the highest ratings in the survey. Communication scored poorly and many respondents suggests there needs to be improved levels of communication in terms of response times and access to support.

The CS profession experiences low levels of interaction with the FTP service. However, those interactions which were recorded in the survey, show some poor experiences for registrants. There also seems to be a lack of advocacy/route for complaints about HCPC from individual registrants. There may need to be more engagement between registrants and their professional body regarding HCPC performance, and more proactivity from the stake-holder, IPEM.

Some of the findings reported here relate to important issues, but the survey data are based on a low response rate. A larger survey across all of Clinical Science is being planned.

### Supplementary Information

**Additional file 1.** The survey questionnaire has been provided as a supplementary file.

## Data Availability

To protect confidentiality of survey respondents, the source data is not available publicly, but are available from the author on reasonable request.

## Abbreviations

AfC: Agenda for Change
AHCS: Academy for Healthcare Clinical Scientists
CPD: Continuous professional development
CE: Clinical Engineer
CS: Clinical Scientist
CoP: College of Paramedics
CT: Clinical Technologist
FoI: Freedom of Information
FTP: Fitness-to-practice
HCPC: Health and Care Professions Council
HR: Human resources
IPEM: Institute of Physics and Engineering in Medicine
JISC: Joint Information Systems Committee
MS: Microsoft
MTO: Medical Technical Officer
PSA: Professional Standards Authority
PSC: Professional Standards Committee
QDA: Qualitative data analysis
UKMPE: UK Medical Physics and Engineering
