# Supplementary material for: Experiences of UK clinical scientists (Physical Sciences modality) with their regulator, the Health and Care Professions Council: results of a 2022 survey
Source: BMC Health Serv Res. 2024 May 16;24:635. doi: 10.1186/s12913-024-10956-7 (PMC11100073; doi:10.1186/s12913-024-10956-7)
Supplement: Supplementary file 1 — Additional file 1. The survey questionnaire has been provided as a supplementary file. [file 12913_2024_10956_MOESM1_ESM.doc]

**Experiences of UK Clinical Scientists & Engineers with their professional regulator - HCPC**

**Introduction**

I am interested in our relationship with the HCPC, as a profession of Clinical Scientists & Engineers, and would like to obtain and analyse a snapshot of our experience with the HCPC and its performance as our regulator. So, I’d be extremely grateful if as many of you as possible could take a little time to complete and respond to this short survey.

If there is a sufficiently good response, I would like to communicate the (anonymised) results directly with the HCPC to congratulate them on positives, and bring to their attention areas for improvement. I not aware of any information of this kind currently available, so I also hope it might be possible to publish the results in SCOPE or a peer-reviewed qualitative journal.

All the information received will be treated confidentially, and stored on a secure network drive. On any shared documentation, identities will be protected, and fully anonymised.
Not all the questions below are compulsory - if you find some don't apply to you, feel free to skip over them. 

Thanks in advance for your help with this.

BW,

Mark


Dr M McJury

Con. Clin. Scientist, Glasgow

1. Alongside answering the questions below, it may be helpful in terms of my understanding, to follow-up on some of the responses in a little more detail. If you would be willing to be contacted for a brief follow-up chat on any queries, please provide an email address.

2. Are you currently on the HCPC register? (Y/N)

3. For how long have you been registered?

4. Please give your post and grade, e.g. Clinical Scientist, B7.

5. Please specify in what UK region you work (i.e. England, Scotland, Wales, or N Ireland).

6. How do you rate the HCPC’s performance, firstly - in terms of value for money?

7. If you can inform/explain your rating above with any specific examples, please do so below.

8. If you are on the HCPC register, how have you found the process of renewing your registration recently?

9. If you didn’t find it easy, could you briefly explain why?

10. How do you rate the HCPC’s handling of your personal data? (e.g. login passwords, etc.)

11. If you are neutral or unhappy about the data handling, can you briefly explain?

12. Have you been audited by the HCPC regarding your CPD? (Y/N)

13. How would you rate this experience?

14. Can you explain your rating above?

15. Have you ever been contacted by the HCPC 'Fitness To Practice' section? (Y/N)

16. How do you rate that experience?

17. Can you explain your rating above?

18. Have you had any other direct interactions with the HCPC? (Y/N)

19. Can you describe what kind of interactions you have had?

20. How do you rate those interactions?

21. If you are neutral or negative about the experience, can you explain why?

22. How do you rate HCPC’s policies and procedures?

23. If you were neutral or negative about policies & procedures, can you explain?

24. Overall, how do you rate the HCPC’s performance as our Regulator?

25. Are there any specific things which might make you improve your rating in the question above?

26. Would you recommend the HCPC as a regulator, to colleagues in a different profession? (Y/N)

27. Finally, if you have any other comments, please add them below.
